# The effects of creatine ethyl ester supplementation combined with heavy resistance training on body composition, muscle performance, and serum and muscle creatine levels

**DOI:** 10.1186/1550-2783-6-6

**Published:** 2009-02-19

**Authors:** Mike Spillane, Ryan Schoch, Matt Cooke, Travis Harvey, Mike Greenwood, Richard Kreider, Darryn S Willoughby

**Affiliations:** 1 Department of Health, Human Performance and Recreation, Baylor University, Box 97313, Waco, TX 76798, USA; 2 Institute for Biomedical Science, Baylor University, Waco, TX 87898, USA; 3 Department of Health and Kinesiology, Texas A&M University, College Station, TX 78743, USA; 4 Interdepartmental Nutrition Program, Purdue University, West Lafayette, IN 47907, USA; 5 Department of Physical Education, United States Military Academy, West Point, NY 10096, USA

## Abstract

Numerous creatine formulations have been developed primarily to maximize creatine absorption. Creatine ethyl ester is alleged to increase creatine bio-availability. This study examined how a seven-week supplementation regimen combined with resistance training affected body composition, muscle mass, muscle strength and power, serum and muscle creatine levels, and serum creatinine levels in 30 non-resistance-trained males. In a double-blind manner, participants were randomly assigned to a maltodextrose placebo (PLA), creatine monohydrate (CRT), or creatine ethyl ester (CEE) group. The supplements were orally ingested at a dose of 0.30 g/kg fat-free body mass (approximately 20 g/day) for five days followed by ingestion at 0.075 g/kg fat free mass (approximately 5 g/day) for 42 days. Results showed significantly higher serum creatine concentrations in PLA (p = 0.007) and CRT (p = 0.005) compared to CEE. Serum creatinine was greater in CEE compared to the PLA (p = 0.001) and CRT (p = 0.001) and increased at days 6, 27, and 48. Total muscle creatine content was significantly higher in CRT (p = 0.026) and CEE (p = 0.041) compared to PLA, with no differences between CRT and CEE. Significant changes over time were observed for body composition, body water, muscle strength and power variables, but no significant differences were observed between groups. In conclusion, when compared to creatine monohydrate, creatine ethyl ester was not as effective at increasing serum and muscle creatine levels or in improving body composition, muscle mass, strength, and power. Therefore, the improvements in these variables can most likely be attributed to the training protocol itself, rather than the supplementation regimen.

## Introduction

Creatine is found in small quantities within the brain, liver, kidneys, and testes, while approximately 95% of creatine stores are found in skeletal muscle [1]. Creatine or methyl guanidine acetic acid is supplied by exogenous sources such as fish and red meat and is endogenously synthesized from the amino acids arginine, glycine, and methionine [2]. Energy is provided to the body from the hydrolysis of ATP into adenosine diphosphate (ADP) and inorganic phosphate (Pi). The phosphagen system provides a rapid resynthesis of ATP from ADP with the use of phosphocreatine (PCr) through the reversible reaction of creatine kinase [2-4]. Of the 95% of creatine stored within skeletal muscle, approximately 40% is free creatine and approximately 60% is PCr [3]. The average 70 kg person has a total creatine pool of 120–140 g. Specifically, the range of creatine in skeletal muscle is 110–160 mmol/kg dry mass [2,1,5]. Creatine supplementation has the ability to increase skeletal muscle stores of creatine and PCr, which could therefore increase skeletal muscle's ability to increase ATP resynthesis from ADP. A previous study [6] employing 20 g of creatine for 6 days showed an increase in PCr concentrations after a maximal isometric contraction during 16 and 32 seconds of recovery. Resistance training along with creatine supplementation has typically been shown to be more beneficial at increasing body mass, maximal strength, and weight lifting performance compared to placebo, but responses are variable [7].

With the ergogenic benefits consistently being shown in both research settings and among the general population, creatine has become one of the most recognized ergogenic aids to date. Intramuscular stores of creatine are considered to be saturated at 160 mmol/kg dry mass; however, only 20% of users achieve this amount and another 20–30% do not respond to creatine supplementation at all [1]. Several hundred studies have examined creatine supplementation's effectiveness in improving muscle performance. Approximately 70% of these studies have shown statistically significant performance improvements, with the remaining studies generally producing non-significant trends [8]. Aside from differences such as experimental design, amount and duration of creatine dosage, training status of participants, etc., the variance in response to creatine supplementation may be due to regulatory mechanisms of a sodium-chloride dependent creatine transporter. The creatine transporter is directly involved in the extracellular uptake of creatine to increase the pool of metabolically active creatine in muscle [9]. It appears that intramuscular creatine uptake is dependent on creatine transporter activity, which has resulted in numerous creatine formulations having been developed in an attempt to improve muscle creatine uptake and potentially increasing the efficacy of creatine supplementation [10].

Due to variations in intramuscular creatine uptake in response to creatine supplementation, it has been suggested that creatine alone may have a limited ability to maximally activate the creatine transporter. Numerous creatine formulations have been developed recently which combine creatine with carbohydrate, sodium, or esterified alcohol with the primary intent of improving cellular absorption and transport which may maximize total intramuscular creatine concentration, thereby improving muscular performance. These new products may prove beneficial increasing creatine uptake by up-regulating or by-passing the creatine transporter. A comparison of creatine monohydrate, creatine with dextrose, and effervescent creatine showed added benefit when dextrose is combined with creatine, but no additional benefits of effervescent creatine compared to creatine monohydrate [11]. Another study combined creatine with magnesium and showed no additional performance benefits compared to creatine monohydrate [12]. Additionally, creatine solubilized in liquid was ineffective at increasing creatine retention compared to creatine monohydrate [8].

The molecular structure of creatine consists of a negatively charged carboxyl group and a positively charged functional group [13]. Creatine is a polar molecule and hydrophilic due to this composition, which limits creatine bioavailability. Esterification is a process widely used by pharmaceutical companies to increase bioavailability of certain prescription drugs with low bioavailability. In a continued attempt to more effectively increase intramuscular creatine levels, one of the latest creatine variations is creatine ethyl ester. Esterification of creatine decreases its hydrophilicity, and is alleged by manufacturers of creatine ethyl ester to by-pass the creatine transporter due to enhanced sarcolemmal permeability toward creatine. However, there are no published data to substantiate this allegation. Furthermore, esterified creatine is unstable in low pH conditions [14,15], and has been shown to be rapidly degraded to creatinine in stomach acid [16]. Even so, manufacturers of creatine ethyl ester claim that it is superior to other forms of creatine, but there is also no published scientific evidence substantiate these claims. Therefore, the effectiveness of creatine ethyl ester has not yet been adequately researched and currently no published data exists to substantiate the alleged effectiveness of this supplement.

The primary purpose of the study was to determine the extent to which creatine ethyl ester affects muscle strength and power, body composition, serum and muscle creatine levels, and serum creatinine levels.

## Methods

### Participants

Thirty apparently healthy males with a mean age of 20.43 ± 1.71 years, height of 176.67 ± 8.02 cm, and total body mass of 80.35 ± 18.52 kg served as participants in the study. The participants were not resistance-trained [not following a consistent resistance training program (i.e. thrice weekly) for at least one year prior to the study], but were recreationally-active. All participants were cleared for participation by passing a mandatory medical screening. Participants with contraindications to exercise as outlined by the American College of Sports Medicine and/or who had consumed any nutritional supplements (excluding multi-vitamins) such creatine monohydrate or various androstenedione derivatives or pharmacologic agents such as anabolic steroids three months prior to the study were not allowed to participate. All eligible subjects signed a university-approved informed consent document. Additionally, all experimental procedures involved in this study conformed to the ethical considerations of the Helsinki Code.

### Testing sessions

The study included baseline testing at day 0, followed by testing sessions at days 6, 27, and 48 in which blood and muscle samples were obtained and where body composition and muscle performance tests were performed.

### Strength assessment

The leg press and bench press maximal strength tests (Nebula, Versailles, OH) were performed by the participants to measure any changes in muscular strength during the course of the study. Four one repetition maximum (1-RM) strength tests were performed during the study at days 0, 6, 27, and 48. Initially, an estimated 50% (1-RM) measured from the previous testing 1-RM test, was utilized to complete 5 to 10 repetitions. After a two minute rest period, a load of 70% of estimated (1-RM) was utilized to perform 3 to 5 repetitions. Weight was gradually increased until a 1-RM was reached with each following lift, with a two-minute rest period in between each successful lift. Test-retest reliability of performing these strength assessments on subjects within our laboratory has demonstrated low mean coefficients of variation and high reliability for the bench press (1.9%, intraclass *r *= 0.94) and leg press (0.7%, intraclass *r *= 0.91), respectively.

### Anaerobic power test

Anaerobic power was determined during each of the four testing sessions at days 0, 6, 27, and 48, and expressed relative to body mass. The determinations were made by performing a 30-second Wingate test on a computerized Lode cycle ergometer (Groningen, Netherlands). A warm-up of 30 rpm for 120 seconds was followed by maximal sprint for 30 seconds against a workload of 0.075 kg/kg of body weight. Correlation coefficients of test-retest reliability of performing these assessments of absolute peak power and mean power on participants within our laboratory has been found to be r = 0.692 and r = 0.950, respectively.

### Body composition assessment

Total body mass (kg) was determined on a standard dual beam balance scale (Detecto Bridgeview, IL). Percent body fat, fat mass, and fat-free mass were determined using DEXA (Hologic Discovery Series W, Waltham, MA). Quality control calibration procedures were performed on a spine phantom (Hologic X-CALIBER Model DPA/QDR-1 anthropometric spine phantom) and a density step calibration phantom prior to each testing session. The DEXA scans were segmented into regions (right & left arm, right & left leg, and trunk). Each of these segments was analyzed for fat mass, lean mass, and bone mass. A sub-region was utilized to determine right thigh mass. The isolated region extended medially to the pubic symphysis down to the head of the femur. Total body water and compartment-specific fluid volumes were determined by bioelectric impedance analysis (Xitron Technologies Inc., San Diego, CA) using a low energy, high frequency current (500 micro-amps at a frequency of 50 kHz). Based on previous studies in our laboratory, the accuracy of the DEXA for body composition assessment is ± 2% as assessed by direct comparison with hydrodensitometry and scale weight.

### Supplementation protocol

Participants were randomly assigned to one of three groups in a double blind manner in which they orally ingested capsules and powder which contained either dextrose placebo [PLC (AST Sport Science, Colorado Springs, CO)], creatine monohydrate [CRT (Integrity Nutraceuticals, Sarasota, FL)], or creatine ethyl ester [CEE (Labrada Nutritionals, Houston, TX)]. For CRT, each capsule contained 250 mg of creatine monohydrate; however, for CEE each capsule contained 700 mg of creatine ethyl ester. Quality control testing of the creatine ethyl ester supplement using NMR from an independent laboratory from the University of Nebraska determined the product to contain 100% creatine ethyl ester HCL, with no detectable creatine HCL or creatinine HCL. The creatine supplement was shown to contain 99.8% creatine monohydrate and 0.2% creatinine.

After baseline testing procedures and fat-free mass determination by DEXA, supplements placebo were ingested relative to fat-free mass based on previous guidelines [17] for 48 days (loading from days 1–5 and maintenance from days 6–48.). Specifically, supplements were ingested at a relative daily dose of 0.30 g/kg fat-free body mass (approximately 20 g/day) during the loading phase, and at a relative daily dose of 0.075 g/kg fat free mass (approximately 5 g/day) during the maintenance phase. After the initial baseline assessment of body composition at day 0, supplement dosages were subsequently adjusted based on body composition assessments performed at days 6 and 27.

In order to standardize supplement intake throughout the study, participants were instructed to ingest the supplements in two equal intervals, one in the morning and one in the evening, throughout the day during the loading phase [13], and at one constant interval, in the morning, during the maintenance phase. Compliance to the supplementation protocol was monitored by supplement logs and verbal confirmation. After completing the compliance procedures the subjects were given the required supplement dosage for the following supplementation period.

### Resistance training protocol

Participants engaged in a 4-day per week resistance-training program split into two upper and two lower extremity workouts per week for a total of seven weeks. The upper body resistance-training program consisted of nine exercises (bench press, lat pull, shoulder press, seated rows, shoulder shrugs, chest flies, biceps curl, triceps press down, and abdominal curls) twice per week and a seven exercise lower extremity program (leg press or squat, back extension, step ups, leg curls, leg extension, heel raises, and abdominal crunches) performed twice per week. We have previously shown this program to be effective at promoting significant gains in muscle strength and mass [18]. Participants performed 3 sets of 8–10 repetitions with 70–80% 1-RM. Rest periods between exercises lasted no longer than three minutes and rest between sets lasted no longer than two minutes. Training sessions were not supervised, but were documented in training logs, and signed off to verify compliance and to monitor progress.

### Muscle biopsies and venous blood sampling

Based on our previously-established guidelines [18], at each of the four testing sessions at days 0, 6, 27, and 48 percutaneous muscle biopsies (50–70 mg) were obtained using a Bergstrom (5 mm) needle. Muscle samples were obtained from the middle portion of the vastus lateralis muscle of the dominant leg at the midpoint between the patella and the greater trochanter of the femur, at a depth between one and two cm. For the remaining three biopsies, attempts were made to extract tissue from approximately the same location as the initial biopsy by using the pre-biopsy scar, depth markings on the needle, and a successive incision that was made approximately 0.5 cm to the former from medial to lateral. After removal, the muscle specimens were immediately frozen in liquid nitrogen and then stored at -80°C for later analysis.

At each of the four testing sessions, venous blood samples were obtained from the antecubital vein using a standard Vacutainer apparatus. Once collected, the samples were centrifuged for 15 minutes. The serum was removed and frozen at -80°C for later analysis. An 8-hour fast prior to blood donation was required for the participants before each of the four testing sessions.

### Muscle and serum creatine analysis

Muscle tissue samples were analyzed spectrophotometrically for total creatine by the diacetyl/*α*-napthtol reaction [19]. Using similar methods, serum samples were measured in duplicate for creatine concentration. Serum samples were immediately ready for creatine analysis, whereas muscle tissue had to first be prepared. For serum creatine analysis, duplicates for all samples yielded a coefficient of variation of 5.4%.

Approximately 10–15 mg of muscle tissue was cut and placed in a microfuge tube, and then placed in a vacuum centrifuge (Savant ISS110 SpeedVac™ Concentrator, Thermo Scientific, Milford, MA) to be spun for 18–24 hours. After sufficient muscle drying, the samples were then placed in an ultra-low freezer at -80°C. Dried muscle was powdered by grinding on a porcelain plate with a pestle. Connective tissue was removed and discarded, whereas powdered muscle was placed into pre-weighed microfuge tubes. Powdered muscle was extracted in a 0.5 M perchloric acid/1 mM EDTA solution on ice for 15-minutes, while periodically vortexing. Samples were then spun at 15,000 rpm at 4°C for 5-minutes. The supernatant was transferred into a microfuge tube and neutralized with 2.1 M KHCO_3_/0.3 M MOPS solution and then centrifuged again at 15,000 rpm for 5-minutes. In order to determine muscle total creatine concentration, supernatant from the above reaction was combined with ddH_2_O and 0.4 N HCl and heated at 65°C for 10-minutes to hydrolyze phosphate groups. The solution was then neutralized with of 2.0 N NaOH and the samples were allowed to incubate at room temperature allowing for color formation, which was detected by a spectrophotometer at 520 nm. Then the samples were run in duplicate against a standard curve of known creatine concentrations. The mean correlation coefficient of variation between duplicates was 1.53%. The standard curve correlation coefficient between plates for total muscle creatine was 0.998.

### Dietary intake records and supplementation compliance

Throughout the course of the study, participants' dietary intake was not supervised; however, it was required that all participants keep detailed dietary records and not change their routine dietary habits throughout the course of the study. As such, participants were required to keep weekly physical activity records and four-day dietary records (three weekdays and one weekend) prior to each of the four testing sessions. The four-day dietary recalls were evaluated with the Food Processor dietary assessment software program (ESHA Research, Salem, OR) to determine the average daily macronutrient consumption of fat, carbohydrate, and protein. The participants were instructed to turn in their dietary records during each testing session. Each participant returned all of their dietary evaluations at the required time points for a 100% compliance rate. In an effort to ensure compliance to the supplementation protocol, participants were supplied with the appropriate amount of supplement to be ingested during the time between last three testing sessions. Upon reporting to the lab for each testing session at days 6, 27, and 48, participants returned the empty containers they had acquired between testing sessions

### Reported side effects from supplements

At the last three testing sessions, participants reported by questionnaire whether they tolerated the supplement, supplementation protocol, as well as report any medical problems/symptoms they may have encountered throughout the study.

### Statistical analysis

Data were analyzed using separate 3 (group) × time [4] univariate analysis of variance (ANOVA) with repeated measures on the time factor with SPSS for Windows Version 16.0 software (SPSS inc., Chicago, IL). Significant differences among groups were identified by a Tukey HSD post-hoc test. A probability level of ≤ 0.05 was adopted throughout.

## Results

### Subject Demographics

Forty-two participants who were initially recruited for the study completed consent forms and participated in an initial familiarization session. Of the 42 participants recruited, 30 completed the 48-day research study. Five participants dropped out due to illness unrelated to the study, five due to apprehension about blood and muscle sampling, and two did not provide specific reasons. However, none of the participants dropped out due to side effects of the supplements or the resistance training protocol. Table 1 shows the sample size, along with the baseline means (± SD) for height, weight, and age for each of the three groups.

**Table 1:** Baseline Participant Demographics

Group	Group Size	Height (cm)	Bodyweight (kg)	Age (yr)
PLA	10	175.39 (7.82)	77.91 (18.44)	20.16 (1.46)

CR	10	173.67 (9.14)	89.45 (22.14)	20.36 (1.53)

CEE	10	177.55 (6.79)	73.75 (14.98)	20.83 (2.21)

### Dietary analysis, supplement compliance, and side effects

All participants appeared to have exhibited 100% compliance with the supplement protocol, and were able to complete the required dosing regimen and testing procedures with no side effects reported from any of the supplements. The diet logs were used to analyze the average caloric and macronutrient consumption relative to total body mass. No significant differences between groups were observed for total kcal (p = 0.901), fat (p = 0.853), carbohydrates (p = 0.871), and protein (p = 0.947). In addition, no significant differences among the four testing sessions were observed for total kcal (p = 0.947), fat (p = 0.956), carbohydrates (p = 0.809), and protein (p = 0.948). This data indicates that there were no significant differences between groups over the course of the study for dietary intake (Table 2).

**Table 2:** Dietary Caloric and Macronutrient Intake

Group/Time	Calories (kcal/kg/day)	Protein (g/kg/day)	Carbohydrate (g/kg/day)	Fat (g/kg/day)
PLA				

Day 0	23.11 (9.29)	1.00 (0.57)	2.88 (1.06)	1.26 (0.485)

Day 6	25.93 (8.94)	1.11 (0.37)	3.29 (1.28)	1.30 (0.421)

Day 27	26.47 (7.14)	1.14 (0.34)	3.96 (1.09)	1.40 (0.501)

Day 48	26.32 (8.34)	1.19 (0.37)	3.24 (1.29)	1.34 (0.293)

CRT				

Day 0	28.49 (9.79)	1.24 (0.50)	3.45 (1.35)	1.38 (0.405)

Day 6	29.67 (9.40)	1.31 (0.27)	3.18 (1.57)	1.43 (0.506)

Day 27	25.86 (8.36)	1.35 (0.38)	3.56 (1.19)	1.41 (0.445)

Day 48	28.43 (9.81)	1.31 (0.47)	3.20 (1.74)	1.51 (0.505)

CEE				

Day 0	21.37 (9.79)	0.94 (0.31)	3.34 (0.82)	1.28 (0.475)

Day 6	19.66 (8.21)	0.97 (0.26)	3.19 (1.12)	1.39 (0.612)

Day 27	18.55 (6.62)	0.86 (0.28)	2.91 (0.95)	1.27 (0.366)

Day 48	17.18 (4.50)	0.79 (0.22)	2.82 (1.22)	1.29 (0.250)

Data are presented as mean (± SD) and expressed relative to total body mass. No significant differences existed between groups or testing sessions for total calories or calories from protein, carbohydrate, or fat (p > 0.05).

### Serum creatine

**Figure 1:**
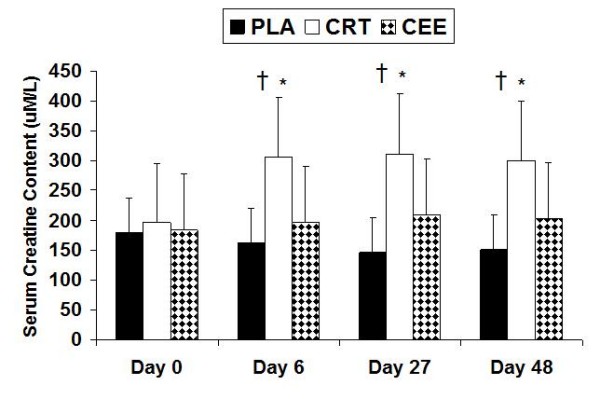
**Changes in serum creatine concentrations with data expressed as mean (± SD)**. † indicates significantly higher serum creatine concentrations in CRT when compared to PLA (p = 0.007) and CEE (p = 0.005). * indicates significant differences for CRT occurred at days 6 (p = 0.028), 27 (p = 0.014), and 48 (p = 0.032).

A significant difference among the three groups was observed indicating significantly higher serum creatine concentrations in the CRT group when compared to PLA (p = 0.007) and CEE (p = 0.005) (Figure 1). Also, significant differences for CRT occurred at days 6 (p = 0.028), 27 (p = 0.014), and 48 (p = 0.032). Muscle creatine

**Figure 2:**
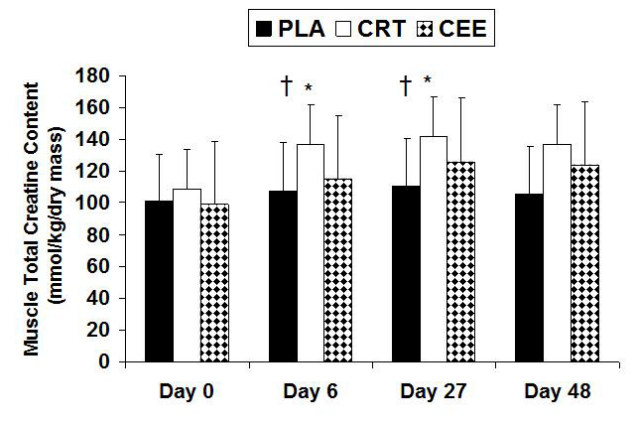
**Changes in muscle total creatine with data expressed as mean (± SD)**. † indicates a significant difference among groups where the PLA group was significantly less than the CRT (p = 0.026) and CEE (p = 0.041) groups. * indicates significant differences over the course of the four testing sessions where CRT increased at day 6 (p = 0.041) and 27 (p= 0.036), and CEE only increased at day 27 (p = 0.043).

A significant difference among groups for total muscle creatine indicated that total muscle creatine content was significantly higher in the CRT (p = 0.026) and CEE (p = 0.041) groups when compared to the PLA group. Significant differences over the course of the four testing sessions were observed indicating that the CRT group underwent increases in total muscle creatine at day 6 (p = 0.041) and 27 (p= 0.036), whereas CEE only increased at day 27 (p = 0.043) (Figure 2).

### Serum creatinine

**Figure 3:**
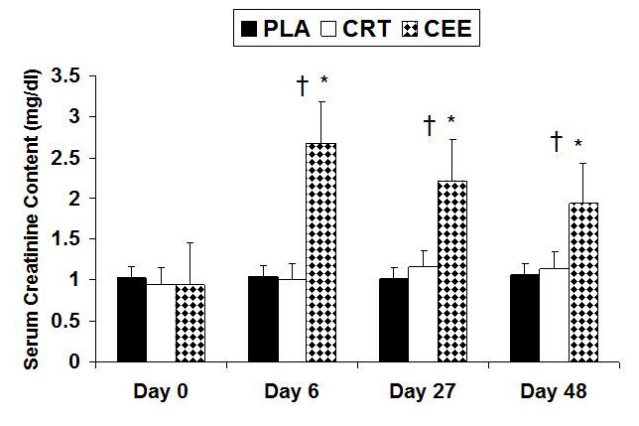
**Changes in serum creatinine with data expressed as mean (± SD)**. † indicates that CEE was greater than PLA (p = 0.001) and CRT (p = 0.001). * indicates significant elevations in CEE at days 6 (p = 0.007), 27 (p = 0.005), and 48 (p = 0.005).

A significant difference over the course of the four testing sessions (p = 0.001) and significant difference between groups (p = 0.001) was observed for serum creatinine. Serum creatinine was greater in the CEE group compared to the PLA (p = 0.001) and CRT (p = 0.001) groups. Further analysis revealed significant elevations in serum creatinine with the CEE group that occurred days 6 (p = 0.007), 27 (p = 0.005), and 48 (p = 0.005) (Figure 3).

### Body composition

There was no significant difference between groups for total body mass (p = 0.173). However, a significant difference over the course of the four testing sessions was observed demonstrating that total body mass significantly increased at days 6, 27, and 48 days 6 (p = 0.015), 27 (p = 0.006), and 48 (p = 0.027) (Table 3). A significant difference between groups (p = 0.043) was observed for fat mass demonstrating that the CRT group had significantly (p = 0.034) more fat mass than the CEE group. Additionally, a significant difference over the course of the four testing sessions was also observed indicating that significant decreases in fat mass were observed at days 6 (p = 0.002), 27 (p = 0.001), and 48 (p = 0.003) (Table 3). For fat-free mass, there was no significant difference between groups (p = 0.137). However, a significant difference was observed among the four testing sessions indicating that fat-free mass significantly increased at days 6 (p = 0.001), 27 (p = 0.001), and 48 (p = 0.001). There was also a significant increase at day 48 compared to days 6 (p = 0.012) and 27 (p = 0.022) (Table 3). There was no significant difference between groups for thigh muscle mass (p = 0.236); however, a significant difference was observed among the four testing sessions which revealed thigh muscle mass to be significantly increased at days 27 (p = 0.017) and 48 (p = 0.016). Increases were also seen at day 27 (p = 0.012) and 48 (p = 0.041) compared to day 6 (Table 3).

**Table 3:** Body Composition Variables

Variables	Day 0	Day 6	Day 27	Day 48
Body Weight (kg)		* (p = 0.015)	* (p = 0.006)	* (p = 0.027)

PLA	77.91 (18.36)	77.94 (17.76)	78.52 (18.64)	78.80 (18.50)

CRT	89.42 (22.08)	90.76 (22.60)	90.55 (22.54)	90.09 (22.86)

CEE	73.69 (14.94)	74.49 (14.48)	74.91 (15.19)	75.32 (15.21)

Fat-Free Mass (kg)		* (p = 0.001)	* (p = 0.001)	* (p = 0.001)

PLA	54.55 (10.05)	55.10 (9.60)	56.05 (10.19)	56.25 (10.22)

CRT	63.27 (10.79)	64.68 (11.18)	65.54 (11.68)	65.12 (11.39)

CEE	59.06 (8.46)	59.74 (8.16)	60.01 (8.52)	60.11 (8.11)

Fat Mass (kg)		* (p = 0.002)	* (p = 0.001)	* (p = 0.003)

PLA	14.34 (8.92)	13.80 (8.65)	13.66 (8.89)	13.68 (8.94)

CRT	21.55 (12.63)	21.09 (12.40)	20.20 (12.06)	20.08 (12.15)

CEE † (p = 0.043)	10.44 (7.28)	10.41 (7.49)	10.50 (7.59)	10.88 (7.88)

Thigh Mass (kg)			* (p = 0.017)	* (p = 0.016)

PLA	8.07 (1.77)	8.17 (1.73)	8.31 (1.73)	8.36 (1.71)

CRT	8.93 (1.78)	9.17 (1.79)	9.28 (1.84)	9.34 (1.93)

CEE	7.58 (.81)	8.06 (1.35)	8.22 (1.31)	8.21 (1.36)

Data are expressed as mean (± SD). * indicates a significant difference at the respective testing session. † indicates a significant difference between groups (p < 0.05).

### Body water

There was no significant difference between groups for total body water (p = 0.276). However, a significant difference existed among the four testing sessions indicating that total body water was significantly increased at days 27 (p = 0.022) and 48 (p = 0.001). There was also a significant increase at day 48 compared to day 6 (p = 0.002) (Table 4). No significant difference between groups existed for intracellular body water (p = 0.198). A significant difference was observed among the four testing sessions indicating there to be increases in intracellular body water at days 27 (p = 0.023) and 48 (p = 0.001). There were also significant increases at day 48 compared to days 6 (p = 0.001) and 27 (p = 0.002) (Table 4). For extracellular body water, there was no significant difference between groups (p = 0.478). A significant difference among the four testing sessions was observed indicating extracellular water to be significantly increased at day 27 (p = 0.042) (Table 4).

**Table 4:** Body Water Variables

Variables	Day 0	Day 6	Day 27	Day 48
Total Body Water (L)			* (p = 0.022)	* (p = 0.001)

PLA	42.36 (8.68)	43.32 (7.86)	44.23 (8.56)	44.79 (7.49)

CRT	46.34 (6.38)	46.74 (6.72)	47.62 (7.16)	48.98 (7.28)

CEE	41.51 (5.77)	42.32 (5.36)	43.11 (6.20)	43.46 (6.10)

Intracellular Body Water (L)			* (p = 0.023)	* (p = 0.001)

PLA	24.90 (5.94)	26.15 (4.77)	26.57 (5.04)	27.42 (4.30)

CRT	27.91 (3.97)	28.19 (3.96)	29.05 (4.53)	30.43 (4.62)

CEE	25.03 (3.98)	24.90 (3.78)	25.87 (4.11)	26.04 (4.03)

Extracellular Body Water (L)			* (p = 0.042)	

PLA	16.94 (3.80)	17.12 (3.30)	17.66 (3.79)	17.36 (3.29)

CRT	18.44 (2.52)	15.56 (2.87)	18.58 (2.71)	18.55 (2.73)

CEE	16.47 (2.06)	17.42 (1.71)	17.25 (2.20)	17.42 (2.24)

Data are expressed as mean (± SD). * indicates a significant difference at the respective testing session (p < 0.05).

### Muscle strength

For bench press strength, no significant difference was observed between groups (p = 0.946); however, a significant difference among the four testing sessions existed indicating that bench press strength was significantly increased at days 27 (p = 0.001) and 48 (p = 0.001). Bench press strength was also significantly increased at day 27 (p = 0.001) and 48 (p = 0.001) compared to day 6, and significantly increased at day 48 compared to day 27 (p = 0.001) (Table 5). No significant difference between groups was observed for leg press strength (p = 0.894). However, a significant difference among the four testing sessions was observed demonstrating that leg press strength increased at days 6 (p = 0.021), 27 (p = 0.001), and 48 (p = 0.001). Increases were also observed at day 27 (p = 0.001) compared to day 6 (Table 5).

**Table 5:** Relative 1-RM Strength Variables

Variable	Day 0	Day 6	Day 27	Day 48
Relative Bench Press Strength			* (p = 0.001)	* (p = 0.001)

PLA	1.04 (.26)	1.10 (.22)	1.12 (.20)	1.15 (.20)

CRT	1.06 (.20)	1.06 (.22)	1.14 (.21)	1.21 (.22)

CEE	1.05 (.28)	1.07 (.30)	1.10 (.29)	1.12 (.29)

Relative Leg Press Strength		* (p = 0.021)	* (p = 0.001)	* (p = 0.001)

PLA	3.55 (.93)	3.70 (.99)	3.90 (.99)	3.83 (.96)

CRT	3.37 (.53)	3.40 (.54)	3.72 (.66)	3.85 (.81)

CEE	3.46 (.71)	3.63 (.72)	3.79 (.67)	3.87 (.72)

Values are represented as means (± SD). * indicates a significant difference at the respective testing session (p < 0.05).

### Anaerobic Power

There were no significant differences between groups for mean (p = 0.468) and peak (p = 0.705) power (Table 4). However, significant differences among the four testing sessions occurred for mean and peak power. Further analysis showed mean power to be increased at days 27 (p = 0.046) and 48 (p = 0.019), along with increases seen at day 48 compared to day 6 (p = 0.029). Peak power was increased at day 48 (p = 0.001). Additionally, peak power was increased at day 48 compared to days 6 (p = 0.001) and 27 (p = 0.029) (Table 6).

**Table 6:** Wingate Muscle Power Variables

Variable	Day 0	Day 6	Day 27	Day 48
Mean Power (W/kg)			* (p = 0.046)	* (p = 0.019)

PLA	623 (136)	633 (154)	636 (166)	657 (177)

CRT	679 (128)	695 (127)	724 (128)	713 (128)

CEE	615 (93)	648 (97)	642 (111)	648 (97)

Peak Power (W/kg)				* (p = 0.001)

PLA	1171 (238)	1197 (313)	1174 (229)	1305 (256)

CRT	1258 (243)	1208 (215)	1322 (214)	1326 (211)

CEE	1107 (202)	1210 (181)	1196 (193)	1251 (174)

Values are represented as means (± SD). * indicates a significant difference at the respective testing session (p < 0.05).

## Discussion

The purpose of this study was to examine the effects of creatine ethyl ester supplementation in combination with heavy resistance training for 47 days compared to supplementation with creatine monohydrate and a placebo. Following a 5-day loading phase and a 42-day maintenance phase, creatine ethyl ester was examined for changes in muscle strength and mass, body composition changes, serum creatine and creatinine levels, and muscle total creatine content.

### Serum and Muscle Creatine

Studies have shown the acute ingestion of 5 g and 20 g of creatine monohydrate to increase serum levels of creatine [5]. The recommended loading and maintenance dosages for creatine ethyl ester are 10 g and 5 g, respectively. As a result, in the present study participants ingested twice the recommended dose of creatine ethyl ester, yet the CRT group resulted in significantly higher levels of serum creatine than the CEE group (Figure 1). Total muscle creatine for the CRT group was significantly greater than the PLA group, but not the CEE group. However, in light of ingesting twice the recommended dose of creatine ethyl ester, total muscle creatine concentration for the CEE group was not significantly different from either the PLA or CRT groups (Figure 2). There was a significant increase in total muscle creatine levels for the CRT at day 6 and 27; however, for CEE an increase was observed to occur at day 27. This is in agreement with most other studies showing significant increases in muscle creatine [3,20-22].

### Serum Creatinine

For serum creatinine, the CEE group underwent significant increases compared to the PLA and CRT groups at days 6 and 48 (Figure 3). In the CEE group, creatinine levels increased 3-fold after the loading phase, and continued to be elevated above normal values throughout the study. This observation can likely be based on the premise that creatine ethyl ester has been shown to be degraded to creatinine in stomach acid (Tallon). Creatinine levels for the CRT group did elevate, but stayed within the normal range of 0.8–1.3 mg/dL, while the PLA group stayed near baseline levels. Serum creatinine is of importance because creatinine is the by-product of creatine degradation. Creatine is non-enzymatically converted into creatinine at approximately 1.7% daily for a typical 70 kg individual [23]. Creatine is also degraded by the gut into creatinine at an estimated rate of 0.1 g of a 5 g dose per hour. This indicates that the GI tract is not a major source of creatinine production; therefore, skeletal muscle is the primary site of creatinine production. [13,24]. With increases in muscle saturation of creatine, creatinine levels will increase due to reduction in the skeletal muscle uptake [1]. In the CRT group, skeletal muscle total creatine content underwent a significant increase at day 6 and 27, whereas the CEE group only increased at day 27. In light of the results for serum creatine and total muscle creatine, based on the premise that serum creatinine levels for CEE were significantly increased at days 6 and 48 (Figures 2 &3) our results seem to indicate that creatine esterification does not provide a superior alternative to creatine monohydrate for muscle creatine uptake.

Supplementation was based on fat-free mass for all groups but was comparable to a 20 g loading phase and a 5 g maintenance phase typically seen with creatine supplementation. When creatine is esterified with an alcohol group, the structure yields approximately 17.4 g of creatine for a 20 g dose and 4.37 g for a 5 g dosage [14]. The recommended loading and maintenance dosages for creatine ethyl ester are 10 g and 5 g, respectively. The supplement loading phase in the present study consisted of two 10 g dosages based on the premise that for a 10 g dose, maximal absorption usually occurs within two hours [13]. Blood draws were not taken specifically after supplementation, yet serum creatinine levels were approximately tripled at day 6 (2.68 ± SD 1.53 mg/dL) compared to baseline (0.95 ± SD 0.18 mg/dL) for the CEE group.

### Muscle Mass and Body Composition

Non-resistance trained participants were selected to perform a 47-day (4 days/week) training program and were expected to have changes in muscle mass and body composition, independent of supplementation. Compared to day 0, all groups showed significant increases in body weight at each of the three testing sessions (Table 3). While all groups increased in total body mass, there was no significant difference between the three groups. Various studies have shown an average of 1–2 kg of total body mass increases with 20 g/day of creatine supplementation for 5–7 days [4,21,23,25]. Total body mass increases after the 5-day loading phase were 0.03 ± 0.60 kg, 1.39 ± 0.46 kg, and 0.80 ± 0.51 kg for PLA, CRT, and CEE, respectively. Kreider [8] indicated that short duration (5–7 days) of creatine supplementation at 20–25 g/day typically leads to increases of up to 1.6 kg in total body mass. The total body mass increase observed with the CRT group was within typical ranges previously seen [26,27], even though there were no significant differences between the groups. For fat mass, fat-free mass, and thigh mass there were no significant differences between any of the three groups. However, collectively fat-free mass was shown to increase at days 6, 27, and 48 compared to day 0. Fat-free mass was also significantly increased at days 27 and 48 compared to day 6 (Table 3). Fat-free mass increases after the 5-day loading phase were 0.55 ± 0.46 kg, 1.41 ± 0.29 kg, and 0.68 ± 0.42 kg for PLA, CRT, and CEE, respectively. Previous studies have shown that longer duration (12 weeks) of creatine supplementation with resistance exercise [28] and shorter duration (5 days loading and 4 days of maintenance) creatine supplementation to increase fat-free mass [29]. As anticipated with an untrained population, increases in body mass and fat-free mass were expected due to a training effect. In line with fat-free mass increases, thigh muscle mass increases were also observed throughout the duration of the study. Thigh mass increases after the 5-day loading phase were 0.10 ± 0.04 kg, 0.24 ± 0.53 kg, and 0.48 ± 0.02 kg for PLA, CRT, and CEE, respectively. In contrast to total body mass and fat-free mass, the CRT group showed the largest increase in thigh muscle mass (Table 3). Fat mass was shown to significantly decrease at days 6, 27, and 48. Both PLA and CRT groups had reductions in fat mass throughout the study, whereas CEE underwent a slight increase (Table 3). Specifically, fat mass was shown to decrease 0.64 ± 0.08 kg and 1.47 ± 0.35 kg, respectively, whereas the CEE group increased 0.44 ± 0.68 kg. Although not statistically significant, it should be noted that the CRT group had a higher baseline fat mass than the PLA and CEE groups. Even though total body mass and fat-free mass were not statistically different, the CRT group may have had a greater potential for reductions in fat mass than the CEE group. As such, the reduction of fat mass observed with the PLA, CRT, and CEE groups was mostly likely due to the resistance training rather than supplementation.

### Body Water

Total, intracellular, and extracellular body water are of particular interest for the CEE group. Claims by the manufactures of creatine ethyl ester have stated a difference in the retention of body water compared to other forms of creatine, specifically creatine monohydrate. Through the use of the esterfication process, creatine is alleged to become more permeable to the sarcolemma and bypass the creatine transporter, thereby allowing more creatine to enter the cell and minimize the amount of extracellular water retained during supplementation. A potential benefit of creatine supplementation is through the action of an anabolic signal for skeletal muscle hypertrophy, with increases in total and intracellular water [5,13]. Roughly two-thirds of the increases in total body water seen during supplementation are intracellular, with no fluid shift occurring [30,31]. Mean increases in total body water (Table 4) from day 0 to day 48 were 2.43 ± 1.19 L, 2.64 ± 0.31 L, and 1.95 ± 0.90 L for PLA, CRT, and CEE groups, respectively. For all groups, total body water was shown to significantly increase at days 27 and 48 compared to day 0. Mean increases in intracellular body water (Table 4) from day 0 to 48 were 2.52 ± 1.63 L, 2.52 ± 0.006 L and 1.01 ± 0.65 L for PLA, CRT, and CEE groups, respectively, and intracellular body water was significantly increased at days 27 and 48. For extracellular water, mean increases from day 0 to 48 were 0.42 ± 0.37 L 0.11 ± 0.18 L and 0.50 ± 0.21 L for PLA, CRT, and CEE groups, respectively, whereas extracellular body water was only significantly increased at day 27 (Table 4).

Collectively, changes in total, intracellular, and extracellular body water were not significantly different between the supplement and placebo groups. However, the mean increases for total and intracellular body water from day 0 to 48 were greatest for the CRT group. Extracellular water increases from baseline were actually largest for the CEE groups. Therefore, claims by the manufactures of creatine ethyl ester stating that extracellular water retention is minimized were shown to be unfounded by the present study. Previous research has shown creatine supplementation to increase total body water, yet no fluid shift occurs [30]. In resistance-trained participants, increases in total body water with creatine supplementation, but not a placebo, during resistance training have been observed [32]. In contrast, in the present study the participants were not resistance-trained, with increases in body water observed in the PLA group. Because resistance training is associated with increases in body water [33], the changes observed in the present study were mostly likely due to the resistance training program itself rather than the supplementation.

### Muscle Strength and Power

Various studies have shown improvements in muscle strength and power through the use of creatine supplementation [1,20,28]. Bench press strength was shown to increase at days 27 and 48 compared to day 0 (Figure 1), whereas leg press strength showed an increase at day 6, 27, and 48 compared to day 0 (Table 5). However, in both instances there were no differences between the three groups. Mean and peak power showed a significant improvement over the course of the study (Table 6). However, the muscle power measures had no significant differences between the three groups. Other studies have shown no benefits for increases in muscle power with supplementation [34].

An increase in muscle performance typically correlates with an increase in creatine muscle uptake [20]. Even though there was no significant increase in total muscle creatine content with the supplement groups over the course of the study. The PLA group, which did not consume creatine, showed similar improvements in muscle strength and performance. Therefore, our data indicates the improvements that were observed were most likely from the strength training program, not due to the creatine supplements.

## Conclusion

Creatine ethyl ester did not show any additional benefit to increase muscle strength or performance than creatine monohydrate or maltodextose placebo. Additionally, total body mass, fat mass, fat-free mass, and thigh muscle mass were not significantly enhanced with creatine ethyl ester supplementation compared to placebo or creatine monohydrate groups. Increases in body water were similar to the placebo and creatine monohydrate groups. The vast majority of the improvement observed in the present study can most likely be attributed to the training protocol itself, rather than the supplementation. Since creatine ethyl ester supplementation showed a large increase in serum creatinine levels throughout the study with no significant increase in serum and total muscle creatine content, it can be concluded that a large portion of the creatine ethyl ester was being degraded within the GI tract after ingestion. Furthermore, it appears that the skeletal muscle uptake of creatine ethyl ester uptake was not significant enough to increase skeletal muscle creatine levels without significant degradation to creatinine occurring.

## Competing interests

The authors declare that they have no competing interests.

## Authors' contributions

MS assisted in coordination of the study, data acquisition, in performing the statistical analysis, and drafting the manuscript. RS, TH, and MC participated in the data acquisition. MG and RK assisted with the design of the study.  RK also secured the donation of the creatine ethyl ester supplement, and assisted in the statistical analysis and manuscript preparation. DSW conceived the study, developed the study design, secured the funding for the project, assisted and provided oversight for all data acquisition and statistical analysis, assisted in drafting the manuscript, and served as the faculty mentor for the project. All authors have read and approved the final manuscript.
